# Supplementary material for: L-form conversion in Gram-positive bacteria enables escape from phage infection
Source: Nat Microbiol. 2023 Jan 30;8(3):387–99. doi: 10.1038/s41564-022-01317-3 (PMC9981463; doi:10.1038/s41564-022-01317-3)
Supplement: Supplementary file 1 — Supplementary Figs. 1–5 and Tables 1–3. [file 41564_2022_1317_MOESM1_ESM.pdf]

nature microbiology

Article

https://doi.org/10.1038/s41564-022-01317-3

L-form conversion in Gram-positive bacteria enables escape from phage infection

In the format provided by the authors and unedited

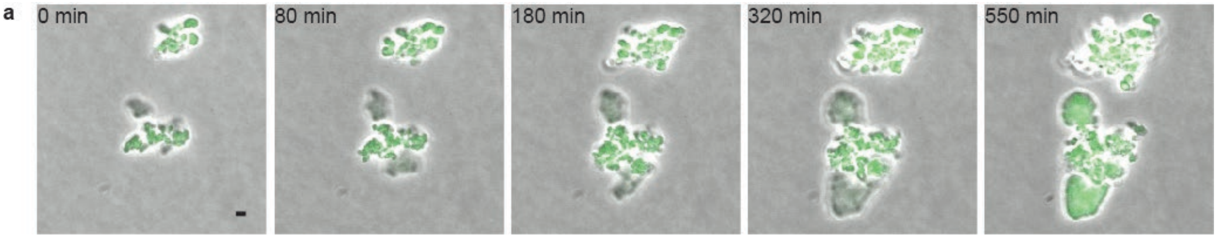

Supplementary Figure 1: Phage infection can trigger L-form switching and proliferation. a, Time-lapse microscopy of proliferating *L. monocytogenes* Rev2 L-forms after L-form switching in response to infection with strictly lytic phage A006 ΔLCR in $DM3_{\Phi}$ medium. Micrograph composites were obtained by merging the PC channel and the channel for green light emission. Individual Frames are extracted from Supplementary Video 2. Scale bar, 2 μm.

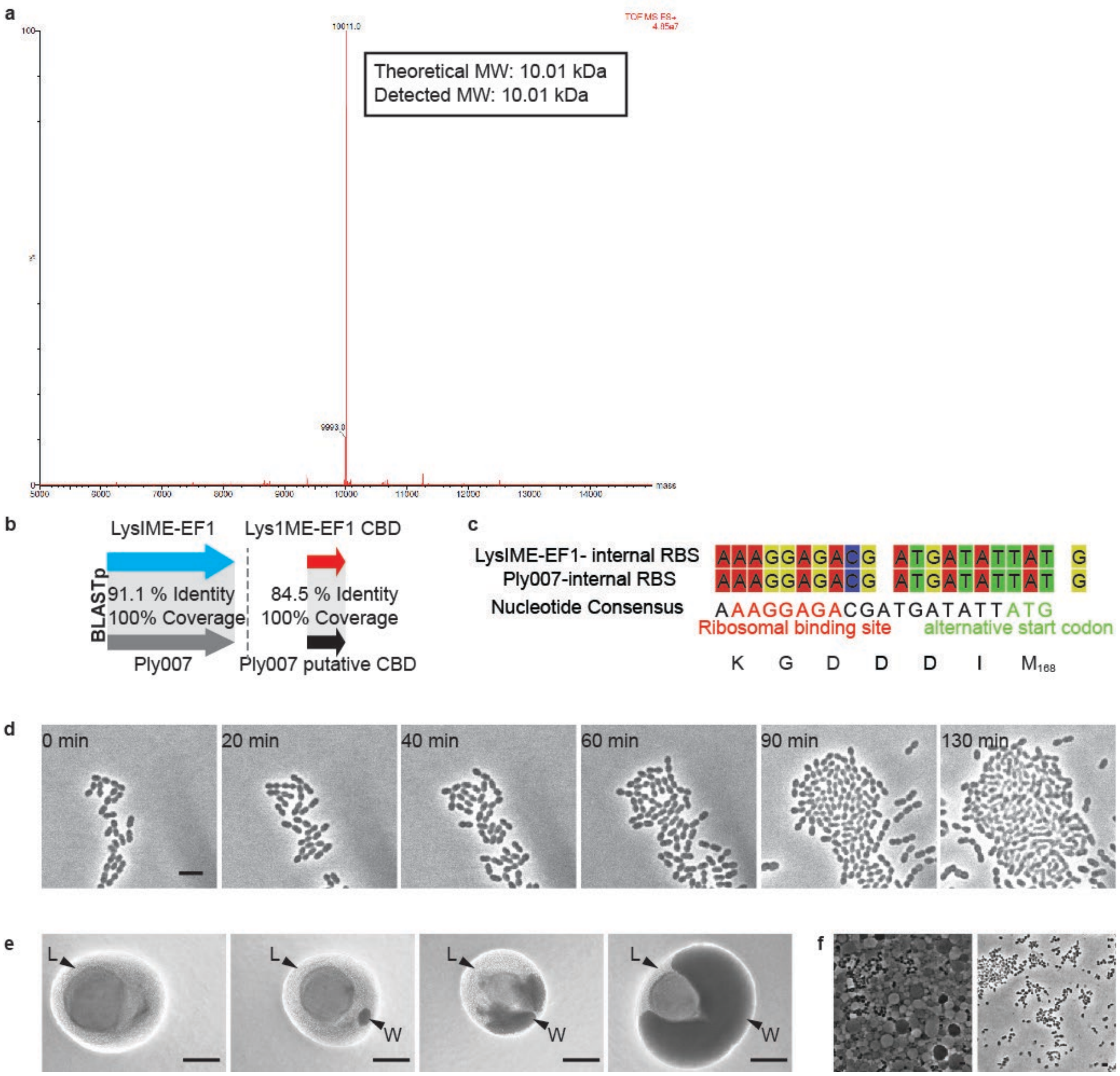

Supplementary Figure 2: L-form switching is promoted by phage-encoded endolysins under osmoprotective conditions. a, A secondary translational product is expressed from ply007. ESI-LC-MS spectra of copurified putative Ply007 peptide fragment (secondary translational product) with fused C-terminal 6xHis-Tag; MW (fragment+Linker+6His-Tag): 10.01 kDa; peptide only: 8.2 kDa. b-c, Ply007 CBD putatively forms a multimeric endolysin together with the corresponding large subunit. b, BLASTp[58] pairwise comparison of Ply007 vs well-characterized LysIME-EF1[59] and Ply007 putative CBD vs LysIME-EF1 CBD. Shown are identity and coverage of gene products (BLASTp) in %. c, Pairwise alignment of the Lys1ME-EF1 internal ribosomal binding site region (RBS) and corresponding region in Ply007. d, Proliferation of walled *E. faecalis* cells on 0.5 BHI-FC agar. e-f, Reversion of *E. faecalis* L-forms 72 h after treatment with Ply007. e, Shown are different bacterial colonies with the L-form phenotype (L) and different stages of reversion to the walled phenotype (W). f, PC micrographs of bacteria obtained from two separate bacterial colonies showing an early-stage reversion phenotype (left) or late-stage reversion phenotype (right). Figures are representative of three independent experiments (e-f). Scale bars,0.5 mm (e), 2 µm (f).

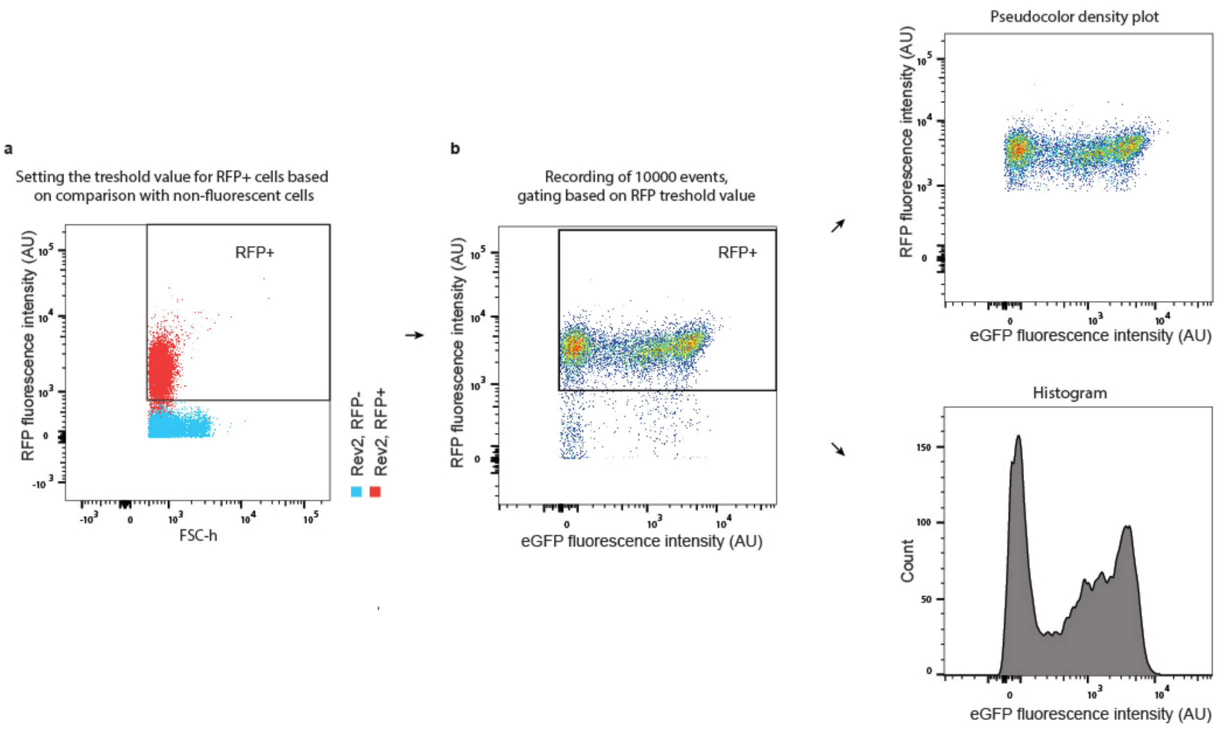

Supplementary Figure 3: Gating Strategy for flow cytometry. a, Identification of bacterial events based on a RFP fluorescence. Treshold values were were set based on comparison of 10000 collected events of non-fluorescent Rev2 cells and cells expressing RFP. b, Shown is one of three replicates of Rev2 cells infected with engineered phage A006::*egfp*$_{cps}$ 75 min post infection. A total of 10000 events was recorded and subsequently gated based on RFP treshold values acquired in (a). Data are displayed as eGFP intensity (AU) vs RFP intensity (AU). The resulting population may be visualized as a pseudocolor density plot (top) or as a histogram (bottom).

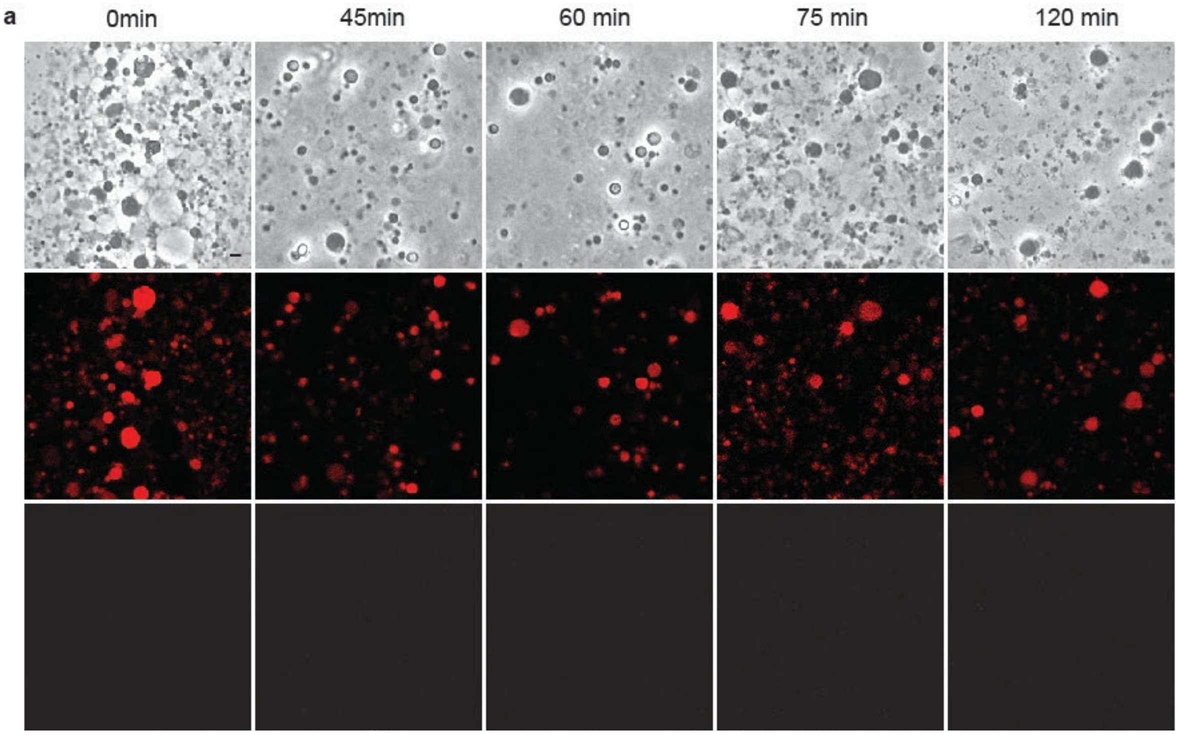

Supplementary Figure 4: Effect of L-form exposure to engineered reporter phages.
a, Rev2 L-forms expressing chromosomally integrated RFP were challenged with excess amounts of phage A006::$egfp_{cps}$ (see also Fig. 5g). Micrographs show samples analyzed at different timepoints from t=0-120 min. eGFP expression was used as a marker to screen for potential phage infection. Note, that no eGFP signal was detectable. PC micrographs and corresponding channels for red (middle panel) and green (bottom panel) light emission are shown. Scale bar, 2 µm.

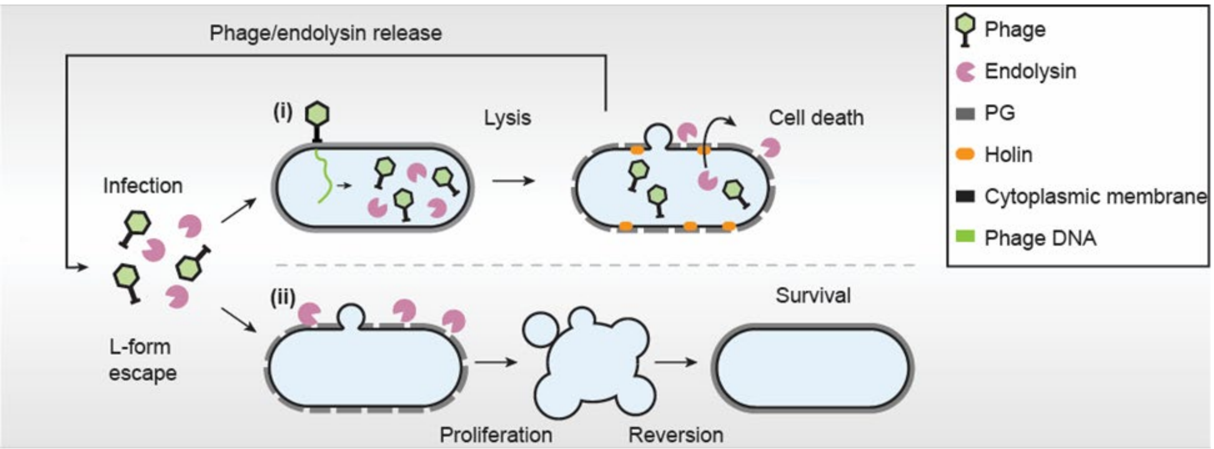

Supplementary Fig. 5: Model for phage-induced L-form escape as a survival strategy. The figure outlines two possible outcomes of an infection with a lytic bacteriophage in a bacterial population. At the time of lysis, infected cells release new phage as well as soluble endolysins. (i) Progeny phages may infect other bacterial cells resulting in lysis and cell death due to the concerted action of endolysins and holins. (ii) Endolysins promote L-form conversion by weakening the cell wall of so far uninfected, walled bystander cells. As a result, wall-deficient L-forms may occur that are completely resistant to phage infection while retaining the ability to proliferate. In absence of selective pressure, L-form cells are capable of reverting to the walled state.

Supplementary Table 1: Strains, phages and plasmids used in this study

Strains, phages, plasmids	purpose/remarks	source/reference
L. monocytogenes EGD-e	-	Lab strain collection, SV 1/2
L. monocytogenes EGD-e *Δlmo1083*	mutant lacking rhamnosylated WTAs	lab strain collection, SV 1/2a
L. monocytogenes EGD-e *Δlmo2550*	mutant lacking GlcNACylated WTAs	lab strain collection, SV 1/2a
L. monocytogenes Rev2	-	*Kilcher et al.*, SV 1/2a
L. monocytogenes Rev2::pPL3/*egfp*	eGFP expression	this work
L. monocytogenes Rev2::pPL2/*rfp*	tagRFP expression	this work
L. monocytogenes Rev2-P100 ("1005")	phage rebooting	this work
L. monocytogenes Mack	propagation host for ΦP35	Lab strain collection, SV 1/2
L. monocytogenes WSLC 1001	propagation host for ΦA118	lab strain collection, SV 1/2
L. ivanovii WSLC3009	propagation host for ΦA511, ΦP40	lab strain collection, SV 5
E. coli BL21 Gold (DE3)	protein expression	Stratagene
E. coli XL1-Blue MRF'	cloning	Stratagene
E. coli XL1-blue MRF' pPL3/*egfp*	plasmid amplification	*Studer et al.*, 2016
E. coli XL1-blue MRF' pPL2/*rfp*	plasmid amplification	*Studer et al.*, 2016
E. faecalis ATCC 19433	-	lab strain collection
E. faecalis Rev	-	this work
ΦA006	-	lab phage collection
ΦA006 ΔLCR	Lysogeny control region deleted --> strictly lytic	*Meile et al.*, 2020
ΦA006::$egfp_{cps}$	eGFP expression	this work
ΦA118	-	lab phage collection
ΦA511	-	lab phage collection
ΦP35	-	lab phage collection
ΦP40	-	lab phage collection

ΦEfs7	-	lab phage collection
pPL2/*rfp*	Cam^R (gram-, gram+), RFP expression	*Studer et al.*, 2016
pPL3/*egfp*	Cam^R (gram-), EryR (gram+), eGFP expression	*Studer et al.*, 2016
pET302	Amp^R, for protein expression	this work
pET302/*ply006*	Amp^R, PlyA006 expression, based on pET 302	this work
pET21a	Amp^R, for protein expression	lab stock
pET21a/*ply007*	Amp^R, Ply007 expression, based on pET21a	this work

*Antibiotic resistances: Cam^R: chloramphenicol, Amp^R: ampicillin, Ery^R: erythromycin

Supplementary Table 2: Sequencing oligonucleotides and DNA strings used in this study.

ID	Sequence (5'-3')	purpose
T7-1	AATACGACTCACTATAGGG	sequencing primer pET302
pET-RP	CTAGTTATTGCTCAGCGG	sequencing primer pET302, pET21a
T7	GAAATTAATACGACTCACTATAGGG	sequencing primer pET21a
JPR1168	TTTAAGAAGGAGATATACATATGGTTAAAGTAAACGATGTAG	pET21a backbone amplification, assembly pET21a/ply007
JPR1169	GCGGCCGCAAGCTTGTCGACTAACTTAACTTGTGGGTAAGC	pET21a backbone amplification, assembly pET21a/ply007
JPR1170	GTCGACAAGCTTGCG	*ply007* amplification, assembly pET21a/ply007
JPR1171	ATGTATATCTCCTTCTTAAAGTTAAAC	*ply007* amplification, assembly pET21a/ply007
String A (*eGFP*, codonoptimized)	ATGTCTAAAGGTGAAGAATTATTCACTGGTGTTGTTC<br>CAATCTTAGTTGAATTAGATGGTGATGTTAACGGTCA<br>TAAATTCTCTGTTTCTGGTGAAGGTGAAGGTGATGCT<br>ACTTACGGTAAATTAACTTTAAAATTCATCTGTACTAC<br>TGGTAAATTACCAGTTCCATGGCCAACTTTAGTTACT<br>ACTTTCGCTTACGGTTTACAATGTTTCGCTCGTTACC<br>CAGATCATATGAAACAACATGATTTCTTCAAATCTGC<br>TATGCCAGAAGGTTACGTTCAAGAACGTACTATCTTC<br>TTCAAAGATGATGGTAACTACAAAACTCGTGCTGAAG<br>TTAAATTCGAAGGTGATACTTTAGTTAACCGTATCGA<br>ATTAAAAGGTATCGATTTCAAAGAAGATGGTAACATC<br>TTAGGTCATAAATTAGAATACAACTACAACTCTCATAA<br>CGTTTACATCATGGCTGATAAACAAAAAAACGGTATC<br>AAAGTTAACTTCAAAATTAGACACAACATTGAAGATG<br>GAAGCGTTCAACTAGCAGACCATTATCAACAAAATAC<br>TCCAATTGGCGATGGCCCAGTTTTATTACCAGATAAC<br>CATTACTTATCTACTCAATCTGCTTTATCTAAAGATCC<br>AAACGAAAAACGTGATCATATGGTTTTATTAGAATTC<br>GTTACTGCTGCTGGTATCACTCATGGTATGGATGAAT<br>TATACAAATAA	construction of phage ΦA006::$eGFP_{cps}$

String B (*ply006*, codon-optimized + *BamHI/NdeI* restriction site)	TATACATATGGCACTGACCGAAGCATGGCTGATTGA<br>AAAAGCAAATCGTAAACTGAATGTGAGCGGCATGAA<br>TAAAAGCGTTGCAGATAAAACCCGCAACGTGATCAA<br>AAAAATGGCCAAAAAAGGCATCTATCTGTGTGTTGCA<br>CAGGGTTATCGTAGCAGCGCAGAACAGAATGCACTG<br>TATGCCCAGGGTCGTACCAAACCGGGTGCAGTTGTT<br>ACCAATGCAAAAGGTGGTCAGAGCAATCATAACTAT<br>GGTGTTGCAGTTGATCTGTGCCTGTATACCAGTGAT<br>GGTAAAAATGTTATTTGGGAAAGCACCACCAGTCGTT<br>GGAAAACCGTTGTTAGCGCAATGAAAGCCGAAGGTT<br>TTGAATGGGGTGGTGATTGGAAAAGCTTTAAAGATTA<br>TCCGCACTTCGAACTGTATGATGCAGCCGGTGGCGA<br>AAAAGCACCGAGCACCAGCGCAAGCAAACCGGCAA<br>CCAGCACCAGCAGTAACAAAAATGTGTATTACACCG<br>AGAATCCGCGTAAAGTTAAAACCCTGGTTCAGTGCG<br>ATCTGTATAATAGCGTTGATTTTACCGAGAAACATAA<br>AACCGGTGGCACCTATCCGGCAGGCACCGTGTTTAC<br>CATTAGCGGTATGGGTAAAACCAAAGGTGGTACACC<br>GCGTCTGAAAACCAAAAGCGGTTATTATCTGACCGC<br>CAACAAAAAGTTCGTGAAAAAAATCTAAGGATCCGGC<br>T	construction of pET302/*ply006*

*Underlined: coding DNA sequences

Supplementary Table 3: Synthetic phage ΦA006::*egfp*$_{cps}$ assembly: Oligonucleotides, templates, and DNA fragments

Fragment ID	Template	ID primer fwd	Sequence (5'-3')	ID primer rev	Sequence (5'-3')	fragment size (nt)
f1	synthetic DNA	JPR668[S]	TCTACAGAAGTCTAAG**AGGAGG**TAAATATATATGTCTAAAGG	JPR669[S]	ATTTCCCTAACCTCCTTATTTGTATAATTCATCCATACCATG	763
f2	A006 gDNA	JPR670	AAGGAGGTTAGGGAAATGCAATTAAAAAAAGAAAATGTCGTTTAC	SK684	GAATATCCTAGCGAATGCGAAATAG	3826
f3	A006 gDNA	SK683	TGAACTATGTCGGTCCTATTTCG	SK686	AATAAATACAATACTTACACCTGGAATG	9294
f4	A006 gDNA	SK685	ATTACAATAGGCCATTCCAGGTG	SK688	CCAAAGATCATCAACAACCATCG	9711
f5	A006 gDNA	SK687	AAACATGGTTGAGAATCCGATGG	SK682	CTTACTGGCAATTTTCACAAGTGG	9294
f6	A006 gDNA	SK681	TTCAGATAAGACAATGCCACTTGTG	JPR671	TATATTTACCTCCTCTTAGACTTCTGTAGAAGCGATTACG	6176

*Bold: RBS inserted via primer, underlined: primer binding site

[S] used for Sanger sequencing of eGFP insert
