# Supplementary material for: L-form conversion in Gram-positive bacteria enables escape from phage infection
Source: Nat Microbiol. 2023 Jan 30;8(3):387–99. doi: 10.1038/s41564-022-01317-3 (PMC9981463; doi:10.1038/s41564-022-01317-3)
Supplement: Supplementary file 2 — Reporting Summary [file 41564_2022_1317_MOESM2_ESM.pdf]

nature portfolio

Corresponding author(s): Martin J. Loessner

Last updated by author(s): Dec 12, 2022

Reporting Summary

Nature Portfolio wishes to improve the reproducibility of the work that we publish. This form provides structure for consistency and transparency in reporting. For further information on Nature Portfolio policies, see our Editorial Policies and the Editorial Policy Checklist.

Statistics

For all statistical analyses, confirm that the following items are present in the figure legend, table legend, main text, or Methods section.

n/a	Confirmed	
☐	☒	The exact sample size (*n*) for each experimental group/condition, given as a discrete number and unit of measurement
☐	☒	A statement on whether measurements were taken from distinct samples or whether the same sample was measured repeatedly
☒	☐	The statistical test(s) used AND whether they are one- or two-sided<br>*Only common tests should be described solely by name; describe more complex techniques in the Methods section.*
☒	☐	A description of all covariates tested
☒	☐	A description of any assumptions or corrections, such as tests of normality and adjustment for multiple comparisons
☐	☒	A full description of the statistical parameters including central tendency (e.g. means) or other basic estimates (e.g. regression coefficient) AND variation (e.g. standard deviation) or associated estimates of uncertainty (e.g. confidence intervals)
☒	☐	For null hypothesis testing, the test statistic (e.g. *F*, *t*, *r*) with confidence intervals, effect sizes, degrees of freedom and *P* value noted<br>*Give P values as exact values whenever suitable.*
☒	☐	For Bayesian analysis, information on the choice of priors and Markov chain Monte Carlo settings
☒	☐	For hierarchical and complex designs, identification of the appropriate level for tests and full reporting of outcomes
☒	☐	Estimates of effect sizes (e.g. Cohen's *d*, Pearson's *r*), indicating how they were calculated

Our web collection on statistics for biologists contains articles on many of the points above.

Software and code

Policy information about availability of computer code

Data collection	cryoET imaging: Tilt series and 2D projection images were acquired using SerialEM 3.7.<br>Data for time course turbidity assays, fluorescence assays, and enzymatic assays were acquired using Omega Software v5.10.<br>Flow cytometry data were collected using Diva 8.01 software (BD Biosciences).<br>Mass spectrometry data were recorded with the MassLynx 4.2 Software (Waters).<br>Microscopic images were acquired using Leica application suite software v. 2.5.1.6757.
Data analysis	Tomogram reconstruction: Drift-correction and exposure-filtering was conducted using Alignframes. Three-dimensional reconstructions and segmentations were calculated using IMOD software package.<br>Flow cytometry: Data analysis was done using FlowJo v10.6.1 (BD Biosciences).<br>Time course turbidity, fluorescence and enzymatic assays: Data analysis and plotting of data was performed in Prism v.8.0 (Graphpad Software).<br>Mass spectrometry: The recorded m/z data were individually deconvoluted into mass spectra by applying the maximum entropy algorithm MaxEnt1 (MaxLynx).<br>Microscopy: Image analysis and processing was performed using Fiji v.1.51.

For manuscripts utilizing custom algorithms or software that are central to the research but not yet described in published literature, software must be made available to editors and reviewers. We strongly encourage code deposition in a community repository (e.g. GitHub). See the Nature Portfolio guidelines for submitting code & software for further information.

Data

Policy information about availability of data

All manuscripts must include a data availability statement. This statement should provide the following information, where applicable:

- Accession codes, unique identifiers, or web links for publicly available datasets
- A description of any restrictions on data availability
- For clinical datasets or third party data, please ensure that the statement adheres to our policy

Representative cryo-tomograms have been deposited in the EMDB database () under accession codes: EMD-16284 – EMD-16291 and EMD-16305 – EMD-16306. Data can be accessed via the Source Data files and Supplementary Tables provided with this paper.

Human research participants

Policy information about studies involving human research participants and Sex and Gender in Research.

Reporting on sex and gender	N/A
Population characteristics	N/A
Recruitment	N/A
Ethics oversight	N/A

Note that full information on the approval of the study protocol must also be provided in the manuscript.

Field-specific reporting

Please select the one below that is the best fit for your research. If you are not sure, read the appropriate sections before making your selection.

☒ Life sciences ☐ Behavioural & social sciences ☐ Ecological, evolutionary & environmental sciences

For a reference copy of the document with all sections, see nature.com/documents/nr-reporting-summary-flat.pdf

Life sciences study design

All studies must disclose on these points even when the disclosure is negative.

Sample size	N/A
Data exclusions	No data were excluded.
Replication	All experiments were conducted at least in biological triplicates.
Randomization	No experimental groups were allocated in this study.
Blinding	Blinding is not applicable for this study.

Reporting for specific materials, systems and methods

We require information from authors about some types of materials, experimental systems and methods used in many studies. Here, indicate whether each material, system or method listed is relevant to your study. If you are not sure if a list item applies to your research, read the appropriate section before selecting a response.

Materials & experimental systems

n/a	Involved in the study
☒	☐ Antibodies
☒	☐ Eukaryotic cell lines
☒	☐ Palaeontology and archaeology
☒	☐ Animals and other organisms
☒	☐ Clinical data
☒	☐ Dual use research of concern

Methods

n/a	Involved in the study
☒	☐ ChIP-seq
☐	☒ Flow cytometry
☒	☐ MRI-based neuroimaging

Flow Cytometry

Plots

Confirm that:

☒ The axis labels state the marker and fluorochrome used (e.g. CD4-FITC).

☒ The axis scales are clearly visible. Include numbers along axes only for bottom left plot of group (a 'group' is an analysis of identical markers).

☒ All plots are contour plots with outliers or pseudocolor plots.

☒ A numerical value for number of cells or percentage (with statistics) is provided.

Methodology

Sample preparation

For analysis of phage-induced eGFP fluorescence, mid-exponential L. monocytogenes strain Rev2 cells expressing RFP cells were diluted to an OD(600 nm) of 0.1. 190 µl of the diluted sample were infected with 10 µl A006::egfpcps 108 PFU at 30°C. Samples were incubated for 45, 60, 75 or 120 min and diluted 1:50 in flow cytometry grade PBS, pH7.4. Diluted samples were immediately analysed from a 1.5 ml tube with no swirling at 4°C.

Instrument

BD FACS Aria III cell sorting device

Software

FlowJo v10.0.6

Cell population abundance

No cell population sorting was conducted.

Gating strategy

Prior to experiments, voltage settings for the relevant fluorescence channels were adjusted by running L. monocytogenes strain Rev2 walled cells expressing no fluorescent proteins or eGFP or RFP. Forward scatter (FSC-H) and side scatter (SSC-H) threshold values were set to 500 to minimize noise. For all experiments, bacterial cells expressing chromosomally integrated RFP were used. Bacterial events were identified based on scatter (FSC-H)- and RFP fluorescence intensity. To eliminate doublets, serial dilutions of bacteria were run to determine the linear range of the event rate

☒ Tick this box to confirm that a figure exemplifying the gating strategy is provided in the Supplementary Information.
