# Supplementary figures and images for: L-form conversion in Gram-positive bacteria enables escape from phage infection
Source: Nat Microbiol. 2023 Jan 30;8(3):387–99. doi: 10.1038/s41564-022-01317-3 (PMC9981463; doi:10.1038/s41564-022-01317-3)

# Source Data Fig. 3

Ply006

Ply007

f

size [kDa]

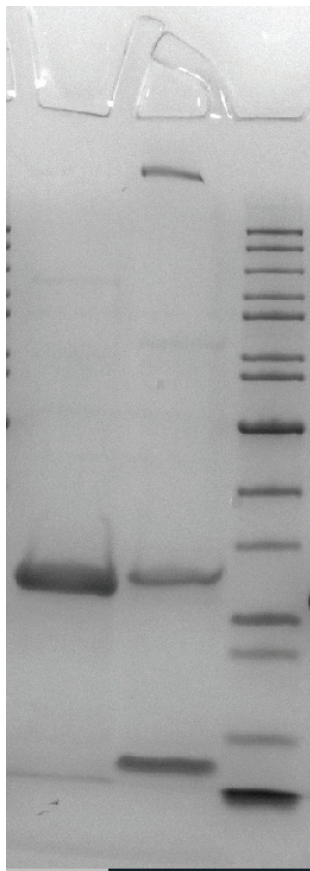

200

50

25

10

Supplement: Source Data Fig. 3 — Full-length, unprocessed gel. [file 41564_2022_1317_MOESM15_ESM.pdf]
